# Response and role of palliative care during the COVID-19 pandemic: A national telephone survey of hospices in Italy

**DOI:** 10.1177/0269216320920780

**Published:** 2020-04-29

**Authors:** Massimo Costantini, Katherine E Sleeman, Carlo Peruselli, Irene J Higginson

**Affiliations:** 1 Azienda USL-IRCCS di Reggio Emilia, Reggio Emilia, Italy; 2 Cicely Saunders Institute, King’s College London, London, UK; 3 Italian Society of Palliative Care (SICP), Milan, Italy

**Keywords:** COVID-19, severe acute respiratory syndrome coronavirus 2, pandemics, hospices, hospice care, palliative care end-of-life care, health care surveys, epidemics

## Abstract

**Background:**

Palliative care is an important component of health care in pandemics, contributing to symptom control, psychological support, and supporting triage and complex decision making.

**Aim:**

To examine preparedness for, and impact of, the COVID-19 pandemic on hospices in Italy to inform the response in other countries.

**Design:**

Cross-sectional telephone survey, in March 2020.

**Setting:**

Italian hospices, purposively sampled according to COVID-19 regional prevalence categorised as high (>25), medium (15–25) and low prevalence (<15) COVID-19 cases per 100,000 inhabitants. A brief questionnaire was developed to guide the interviews. Analysis was descriptive.

**Results:**

Seven high, five medium and four low prevalence hospices provided data. Two high prevalence hospices had experienced COVID-19 cases among both patients and staff. All hospices had implemented policy changes, and several had rapidly implemented changes in practice including transfer of staff from inpatient to community settings, change in admission criteria and daily telephone support for families. Concerns included scarcity of personal protective equipment, a lack of hospice-specific guidance on COVID-19, anxiety about needing to care for children and other relatives, and poor integration of palliative care in the acute planning response.

**Conclusion:**

The hospice sector is capable of responding flexibly and rapidly to the COVID-19 pandemic. Governments must urgently recognise the essential contribution of hospice and palliative care to the COVID-19 pandemic and ensure these services are integrated into the health care system response. Availability of personal protective equipment and setting-specific guidance is essential. Hospices may also need to be proactive in connecting with the acute pandemic response.

**What is already known about the topic?**

- The Coronavirus disease 2019 (COVID-19) has estimated global mortality of 3.4%, and numbers of cases are rapidly escalating worldwide.
- Hospice services face unprecedented pressure, with resources rapidly stretched beyond normal bounds.
- No data exist on the response and role of hospice and palliative care teams to COVID-19.
- Within Europe, Italy has been most affected by COVID-19.

**What this paper adds?**

- We surveyed 16 Italian hospices in March 2020 to better understand the response to COVID-19 by palliative care in a rapidly evolving situation.
- Hospices had implemented rapid policy and practice changes in response to COVID-19, which included moving to more support in community settings, change in admission criteria and daily telephone support for families.
- Personal protective equipment were inadequate, limiting the hospice response.
- Setting specific guidance was lacking, limiting the response.
- Assessments of risk and potential impact on staff varied greatly.

**Implications for practice, theory or policy**

- Governments must recognise the hospice and palliative care sector as an essential component of the healthcare system response to COVID-19.
- The hospice sector is capable of responding rapidly to the COVID-19 pandemic, but the potential of this response will be undermined unless hospices can access personal protective equipment, and there is attention to sector specific guidance and support.
- Considerations for hospice services during the COVID-19 pandemic are changes to visitor policies, interruption of volunteering, shifting roles and responsibilities such as greater community working and telephone support for relatives.

## Introduction

Coronavirus disease 2019 (COVID-19), caused by severe acute respiratory syndrome coronavirus 2 (SARS-CoV-2), emerged in Wuhan, China, in December 2019. On 11 March 2020, COVID-19 was declared a pandemic by the World Health Organization (WHO). The WHO estimates global mortality at 3.4%,^1^ though mortality rates are higher among older people and those with comorbidities. A cohort of 36 non-survivors of COVID-19 in China identified the most prevalent symptoms as fever (94%), shortness of breath (58%), fatigue (47%) and cough (39%).^2^

Within Europe, Italy was the first country to be seriously affected by COVID-19. The first case was identified on 21 February 2020, and by the end date of this survey, on 15 March 2020, more than 24,000 cases and 1,809 deaths were recorded in Italy. Escalating numbers of deaths are anticipated elsewhere. Palliative care is an essential component of health care in pandemics, contributing to symptom control, as well as psychological support for patients, carers and health care professionals, and supporting triage and complex decision making.^3^ However, hospices may be particularly vulnerable to disruption in pandemics, and very little data exist on the response of, or impact on, palliative care services in these situations.^4^

The aim of this study was to examine preparedness for, and impact of, COVID-19 on hospices in Italy. In particular, we wanted to understand the early experiences, as these may help to inform the response in other countries who were about to experience the challenges of Italy. Our research questions were as follows:

- How were hospices changing their procedures and guidance as a result of the COVID-19 pandemic, including dealing with positive cases among patients and staff?
- What impact did COVID-19 have on their staff and how much were they concerned about staff being infected?
- What aspects concerned them or limited their ability to respond to COVID-19?
- Were there changes in their practices and services in response?

We also wanted to determine any experience of COVID-19-positive patients and whether this affected their response. We explored whether hospices in areas of higher and lower of COVID-19 population prevalence led to differences in response, to see whether this appeared to lead to greater preparation in areas first to be affected and with more cases or other differences in response.

## Method

### Design

A telephone survey of a sample of Italian hospices was conducted.

### Population/setting

Italy has around 570 palliative care services, serving its population of just over 60 million people, with 0.9 services per 100,000 inhabitants. Services include inpatient hospices, home care services and dedicated wards and teams in hospitals, sometimes linked to hospices.^5^ Almost all are either managed by the public sector or are charitable, that is, private not for profit. The density of services is higher in the north of the country, including Lombardy, where the main COVID-19 outbreak began, than in the south. There is a long tradition of hospice and palliative care: the European Association for Palliative Care (EAPC) was founded in Italy in 1988. There are however few academic positions and little education in medical schools. The EAPC 2019 Atlas of Palliative Care recorded that 0 of 43 medical schools and only 98 of 222 nursing schools provided palliative care education within their undergraduate programmes.^5^

### Sampling

We purposively sampled hospices from regions according to the number of COVID-19-positive patients per 100,000 inhabitants: low (<15 cases), medium (15–25 cases) and high (>25 cases), with a slight oversampling of high prevalence areas. We included a mix of public and private/not for profit hospices and different sizes (e.g. number of beds). We chose purposive sampling as a non-probability sample method, with our selection based on characteristics of a population and our research questions.

### Recruitment

Hospices were contacted by telephone by M.C. or C.P. between 11 and 15 March 2020, and if they agreed to take part an interview with the medical or nursing director was requested and arranged for a suitable time.

### Data collection

Interviews took place by telephone. We deliberately kept the questionnaire brief because of anticipated time pressures of the respondent. Its content was developed based on our research questions to guide the interviews (Supplemental Appendix 1). We included the opportunity for free text comments (Supplemental Appendix 1). The questions were read to respondents and the interviewer wrote down their response verbatim. A Likert-type scale was used to understand perceptions of risk (0 = no risk, 10 = maximum risk imaginable). All the data were translated into English before analysis and all authors had full access to the data.

### Analysis

Descriptive analysis was undertaken of quantitative and qualitative data. We explored patterns and did not plan statistical comparisons.

### Ethical issues

The ethics committee of Reggio Emilia was approached, and we were advised that according to Italian law a formal ethical approval was not necessary. We agreed with hospices to keep their identity anonymous. We were conscious that the interview may be distressing for hospice directors, and so we stressed that participation was voluntary and included open comments for them to say what else was of concern to them.

### Sample size

We continued to sample hospices until data reached saturation and no new themes were emerging.

## Results

All hospices we approached agreed to take part and provided data. We included 16 hospices: size ranged from 7 to 34 beds (mean = 15.9; standard deviation (SD) = 8.3) (Table 1). Four were in low prevalence areas, five were in medium prevalence areas and seven were in high prevalence areas. Interviews were conducted with 13 medical directors and three directors of nursing. Eleven hospices were public and five were private (not for profit). Seven hospices were affiliated with acute hospitals, including two hospitals that had been designated exclusively COVID-19-positive hospitals. Two hospices in the ‘high’ COVID-19 prevalence area had experienced cases of COVID-19 positivity. Cases included both patients and staff (including nurses, physicians and health care assistants). Interviews lasted between 10 and 15 min.

**Table 1.** Characteristics of the hospices that provided data and their rating of effects on workforce and risk.

Hospice ID	Area	Hospice characteristics			COVID-19-positive cases	Effects on workforce (Likert scale 0–10)^a^		Perception of risk (Likert scale 0–10)^a^	
		Number of beds	Hospice type	Hospital association		Anxiety about needing to care for children	Anxiety about needing to care for relatives	Of staff becoming unwell	Of hospice closure
1	Low prevalence	10	Public	Yes	No	5	5	2	1
2		25	Private non-profit	No	No	8	3	0	0
3		7	Public	No	No	7	8	5	2
4		10	Public	Yes	No	6	6	3	0
5	Medium prevalence	11	Public	No	No	6	8	4	0
6		12	Public	Yes	No	3	3	5	3
7		34	Private non-profit	No	No	6	3	6	2
8		10	Public	Yes	No	5	4	9	5
9		29	Public	Yes^b^	No	10	10	7	0
10	High prevalence	30	Private non-profit	No	No	8	8	6	3
11		10	Public	Yes	Yes	7	7	3	0
12		16	Public	No	No	6	6	5	4
13		16	Private non-profit	No	Yes	1	3	2	2
14		12	Private non-profit	No	No	6	6	8	7
15		10	Public	Yes	No	7	7	1	0
16		12	Public	No	No	3	3	3	1

a Scale was 0 = no risk, 10 = maximum risk imaginable.

b This unit includes eight beds that are affiliated to a hospital.

### Procedures and guidance

Most hospices agreed that there was written guidance covering procedures in the event that patients, relatives or staff members either tested positive for COVID-19 or were suspected cases. All hospices followed national and regional guidance, in six cases guidelines of the local main hospital. The guidelines described were primarily regarding the procedures for the management and notification of COVID-19-positive people. None used guidelines or procedures specific for hospices though in one case guidance was described as locally defined but ‘strengthened’ for the hospice. No hospices had written guidance that referred specifically to volunteers, and 15 of 16 stated that they were no longer using volunteers.

### Personal protective equipment

Equipment including gloves, masks and disposable gowns were being used, though use and provision varied considerably. One respondent in a high prevalence area noted use of ‘very rigorous dressing procedures including FP2 masks and special overalls’, while in another high prevalence area the interviewee commented, ‘no mandatory protection, professionals can choose what to do’. A third interviewee (high prevalence area) noted use of ‘gloves and masks . . . but there is a great scarcity of this equipment’. Other comments included ‘some FP2 masks are available but they are never used’ (two respondents); ‘FP2 masks only for patients with respiratory symptoms’ (two respondents); ‘gloves, masks, all body covered’; ‘surgical masks for patients and manoeuvres at risk’; and ‘total protection: mask, disposable gown, gloves’.

### Changes in visiting policies

All hospices had changed visitor policies, though there was not a unified approach to this. Twelve had adopted a policy of allowing only one relative per patient. Two of these hospices (high prevalence areas) were willing to relax this policy when patients where dying, while one (medium prevalence area) only allowed visitors when patients were dying. One hospice (high prevalence area) required that visitors needed to remain in the hospice day and night and that they could not return once they had left the hospice, and two (high and medium prevalence areas) had completely closed to visitors. One hospice (medium prevalence area) comprised two separate units, one of which had adopted a ‘one visitor only’ policy, and the other had closed to visitors. Two hospices (medium prevalence areas) were screening relatives for symptoms before entering the hospice. One hospice described having locked the door of the hospice unit, with limited access only via a bell. Of the limited visiting hours, one interviewee (high prevalence area) noted, ‘relatives seem to understand what the staff are doing and appreciate their work’.

### Admission criteria

Most hospices reported no change in their admission criteria, though one had cancelled respite admissions and closed to admissions from hospitals. Three hospices had implemented a telephone triage system before admission to assess risk of COVID-19 positivity. Two hospices (high prevalence areas) were openly accepting patients infected with COVID-19, who were being isolated in specific areas of the hospices. One hospice (high prevalence area) had a policy not to admit patients known to have COVID-19 at the time of the survey. They were continuing to admit their existing patients. Another hospice (high prevalence area) had observed a reduction in the number of requests for admission to hospice. Several hospices said they were liaising and working flexibly with other hospice and palliative care services.

### Care after death

Care after death varied, with four hospices limiting the number of relatives who could view the body of the deceased patient. One hospice (high prevalence area) had banned any relatives from entering the mortuary, and another (high prevalence area) had adopted a system where relatives viewed the body of the deceased through a window.

### Impact on workforce

Hospices reported moderate levels of staff anxiety about the need to care for either children (mean response 5.4 on Likert-type scale 0–10) or other relatives (mean response 5.7), and there was little pattern between high, medium and low prevalence areas. Several hospices reported that staff were worried about coming to work, for example, one nurse coordinator (medium prevalence hospice) reported, ‘staff are very worried and agitated about the risk to themselves and the possibility of taking the virus home’. Nevertheless, staff absence was low, with one physician commenting, ‘the staff are scared but still they go to work’.

### Assessment of risk

Hospices perceived a moderate risk of hospice staff being infected with COVID-19 over the coming week (mean response 4.0 on Likert-type scale 0–10). This was higher in medium (6.2) and high (4.0) prevalence areas than in low prevalence areas (2.5). Hospices in low prevalence areas perceived a low risk of the hospice closing in the coming week because of infected staff members (mean response 0.75 on Likert-type scale). This was greater in medium (2.0) and high prevalence (2.4) areas though remained low.

### Changes in practice

Several hospices had rapidly implemented changes in practice. One hospice (high prevalence area) had noted a reduction in requests for admission so had moved staff from inpatient to home care services. Another (high prevalence area), where visiting had been severely limited, had implemented a system where the hospice psychologist was telephoning patients’ relatives every day to update them and provide psychological support. Other hospices had cancelled all internal meetings, as well as annual leave.

### Psychological impact on staff

A lack of adequate preparation made caring for COVID-19-positive patients difficult: one medical director noted, ‘Positive patients entered and we were not prepared’.

### Other comments

Several interviewees spoke of the difficulty of providing holistic care within the constraints of an infectious disease outbreak: ‘It is difficult to maintain the humanity of palliative care in this situation’. This included the acute setting ‘Guidance on care for people dying from COVID-19 is missing’, while one physician noted, ‘People with this infection are dying in ICU very badly, without any kind of palliative care support’. The impact of COVID-19 on care of the dying was felt to reach beyond the acute illness: ‘At the end of this story, I think palliative care in Italy and everywhere will be very different from before’.

## Discussion

### Main findings

We provide the first data from the hospice sector of preparedness for, and impact of, COVID-19. At the time of data collection, two hospices (both in high prevalence areas) were aware of having COVID-19-positive patients or staff. However, all had implemented changes in policies in response to COVID-19, for example, concerning visitors and volunteers, and several had rapidly implemented changes in practice according to changing needs.

An important concern voiced by staff was a lack of preparedness for COVID-19. While all hospices in our survey had written guidance on procedures for suspected and confirmed cases of COVID-19, this was mostly regionally or nationally provided and no hospice had setting-specific guidance. There was considerable variation in the use of barrier precautions and personal protective equipment, which were described as scarce. There was also wide variation in perceptions of anxiety and the risk of illness among staff. This may indicate that more and urgent education is needed to inform hospice staff about reducing risks of COVID-19 infection. Protection of health care professionals across all settings against COVID-19 through use of appropriate barrier precautions should be of the highest priority, to avoid illness and mitigate against psychological distress.^1^ National and international guidance and evidence regarding personal protective equipment has been evolving since the start of this pandemic. A recent rapid review concluded that the best evidence was that ‘Suspected or confirmed case of COVID-19 requiring healthcare facility admission and no [aerosol generating procedures] AGPs, use hand hygiene, mask, gown, goggles, gloves’. This review noted that masks are one component of protection. Aprons, gloves and eye protection should all be used with confirmed or suspected COVID, something which has been recommended but used variably across the globe.^6^

Provision of holistic care in the context of an infectious disease outbreak was noted to be highly challenging. Hospices responded to this challenge through rapid changes to service provision. For example one hospice had implemented daily telephone calls to relatives who were unable to visit, which might mitigate against the ‘disruption in connectedness’ described following the 2003 SARS epidemic in Singapore.^7^ Another hospice had moved staff from inpatient to home care services as a result of a falling number of inpatient referrals. Changes in inpatient hospice utilisation in Taiwan were identified during and after the SARS epidemic and led to recommendations to distribute hospice care services into networks (e.g. home care, acute hospital inpatient care and inpatient hospice care) that can adapt to changing needs.^4^ The concern regarding palliative care for many patients who the services could not reach was a pervading theme, as seen in the other comments. The engagement of palliative care in intensive care can be complex, often because of the uncertainly in outcome and the rapidly changing situation.^8^ To date, attention has focussed on tools and integrated working to support patients and families in intensive care unit (ICU).^9,10^

### Strengths and limitations

The limitations of this study are that it was a rapid telephone survey with a small sample. We chose purposive sampling as this is known to be very useful in situations when a targeted sample is needed quickly, and where sampling for proportionality is not the main concern, as in the context of rapid spread of COVID-19 and a lack of information on the hospice response. Although it would have been useful to have had a larger sample, we reached saturation of themes. However, a larger sample might have identified more examples of innovate practice. The opportunity to collect in-depth qualitative data was limited due to the extreme pressure services are under. It will be useful to follow up this cross-sectional survey over time, and with a larger sample, as the situation changes. Using our existing clinical-academic networks enabled this survey to be completed rapidly, to provide essential early data of the hospice response.

### What this study adds

The hospice sector has an important role to play in the response to COVID-19. Hospices are known to provide support with complex decisions and triage, psychological support for patients, carers and professionals, and complex symptom management, particularly for people who are dying.^3^ However, in our study we found that procedures had mainly focussed on the notification regarding positive COVID-19 cases, rather than on the contribution of hospices to care. There is other evidence that the hospice sector is underused in epidemics.^4^ In Italy, and elsewhere, it is likely that the number of people dying with COVID-19 will overwhelm the capacity of the acute sector.^11^ Integrating hospice and palliative care into the acute pandemic response may improve the care and symptom management for people who are deteriorating and at the end of life, as well as helping in the overall effort to optimise survival of others.^12^

Our data highlight that hospice services in all countries need to act now to prepare for COVID-19. Building on the Critical Care model of providing surge capacity in a crisis, elements essential to implementing a palliative care pandemic plan include (a) medication and equipment for symptom control including kits for use in care homes and at home; (b) education to frontline staff on symptom management and end-of-life care including developing standardised order sheets and protocols, and involving allied care workers in providing psychological and bereavement support; (c) identification of wards and beds appropriate to accommodate patients expected to die; and (d) systems to identify patients in need of palliative care and to provide appropriate support across settings.^13^

## Conclusion

Hospices are uniquely placed to rapidly develop expertise in holistic care for people with COVID-19, including direct care of the dying as well as facilitating advance care planning in anticipation of acute deterioration. Our survey demonstrates that the hospice sector is able to respond flexibly and rapidly to the COVID-19 pandemic. However, the potential of hospices in supporting the COVID-19 pandemic will be undermined unless the sector has access to appropriate protective equipment and setting-specific guidance. Governments must urgently recognise the necessity of hospice and palliative care to the COVID-19 pandemic and ensure these services are both protected and integrated into the health care system response. Hospices may also need to reach out to offer support in creative ways during the response.

## Supplemental Material

interview_4.0_clean-1 – Supplemental material for Response and role of palliative care during the COVID-19 pandemic: A national telephone survey of hospices in Italy Click here for additional data file. Supplemental material, interview_4.0_clean-1 for Response and role of palliative care during the COVID-19 pandemic: A national telephone survey of hospices in Italy by Massimo Costantini, Katherine E Sleeman, Carlo Peruselli and Irene J Higginson in Palliative Medicine
