# Supplementary material for: Response and role of palliative care during the COVID-19 pandemic: A national telephone survey of hospices in Italy
Source: Palliat Med. 2020 Apr 29;34(7):889–95. doi: 10.1177/0269216320920780 (PMC7218350; doi:10.1177/0269216320920780)
Supplement: interview_4.0_clean-1 – Supplemental material for Response and role of palliative care during the COVID-19 pandemic: A national telephone survey of hospices in Italy [file interview_4.0_clean-1.pdf]

THE INTERVIEW
(version 4.0)

INTERVIEWER: **☐ Carlo Peruselli** **☐ Massimo Costantini**
DATE: ………………………….
INTERVIEVED: **☐ Physician Responsible** **☐ Nurse Responsible** **☐ Nurse coordinator**

HOSPICE CHARACTERISTICS

- Number of beds …………..
- ☐ Private / nonprofit ☐ public
- ☐ Within a hospital ☐ Not within a hospital

2. CASES OF POSITIVITY for COVID-16 (tick)

☐ patients
☐ relatives
☐ physicians
☐ nurses
☐ volunteers
☐ others (specify) …………………………………………….

If YES (for at least one)
Describe who was positive (by group), how identified, what was done, what were the consequences

3. PROCEDURES (written guidance)
3.1 Do you have a written procedures for "what to do" for <u>a positive case</u> among

Patients or relatives	☐ yes	☐ no
Professional staff member	☐ yes	☐ no
Volunteers	☐ yes	☐ no

3.0 Do you have a written procedures for "what to do" for a <u>suspected positive case</u> among

Patients or relatives	☐ yes	☐ no
Professional staff member	☐ yes	☐ no
Volunteers	☐ yes	☐ no

4. OTHER PROCEDURES (measures to avoid contagion)
Which measures did you take to avoid contagion?

4.1 Modification of policy for visitors / relatives (number of visitors, hours etc.)
4.2 Operator protection (Personal Protective Equipment)
4.3 Modification of policy for patients' admission to the hospice
4.4 Modification of volunteer support policy
4.5 Modification of policy regarding care of the relatives after the patient's death

5.0 Did you take the measures of point 4 and 5 spontaneously or did you receive instructions from health management or regional authorities?

6. EFFECTS ON STAFF
6.1 Did you observe unjustified absences of the staff?
6.2 Were staff anxious about the need to care for children who were not at school?
Likert Scale 1-10
6.3 Were staff anxious about the need to care for their own relatives?
Likert Scale 1-10

7. PERCEPTION OF THE RISK
In the coming week ….
7.1 How much do you think hospice staff are at risk of being infected?
Likert scale 0-10 (0 no risk - 10 maximum risk you can imagine)

In the coming week ….
7.2 How much do you think the hospice is at risk of closing because of infection of hospice staff?
Likert scale 0-10 (0 no risk - 10 maximum risk you can imagine)

6. Do you think there are relevant information we have omitted to ask you?
